# Rheumatology-led pregnancy clinic: enhancing the care of women with rheumatic diseases during pregnancy

**DOI:** 10.1007/s10067-020-05173-6

**Published:** 2020-06-03

**Authors:** Yasser El Miedany, Deborah Palmer

**Affiliations:** 1 H. Senior Clinical Lecturer, King’s College, London, UK; 2 Rheumatology Department, North Middlesex University Hospital, London, UK

**Keywords:** Autoimmune rheumatic diseases, Contraception, Counselling, Family planning, Pregnancy, Psoriatic arthritis, Rheumatoid arthritis, Rheumatology nurse, Systemic lupus erythematosus, Women’s health

## Abstract

The autoimmune rheumatic diseases have a clear predilection for women. Consequently, issues regarding family planning and pregnancy are a vital component of the management of these patients. Not only does pregnancy by itself causes physiologic/immunologic changes that impact disease activity but also women living with inflammatory arthritic conditions face the additional challenges of reduced fecundity and worsened pregnancy outcomes. Many women struggle to find adequate information to guide them on pregnancy planning, lactation and early parenting in relation to their chronic condition. This article discusses the gaps in the care provided to women living with inflammatory arthritis in standard practice and how a rheumatology nurse-led pregnancy clinic would fill such gap, consequently enhance the care provided and ensure appropriate education is provided to these individuals who represent the majority of the patients attending the rheumatology outpatient clinics. Such specialist care is expected to cover the whole journey as it is expected to provide high-quality care before, during and after pregnancy.

## Introduction

Autoimmune rheumatic diseases (ARDs), in particular systemic inflammatory rheumatic diseases which include rheumatoid arthritis [RA], systemic lupus erythematosus [SLE], ankylosing spondyloarthritis [AS], antiphospholipid syndrome [APS] and systemic sclerosis, are lifelong, autoimmune systemic diseases more prevalent in women of childbearing age, who are diagnosed in their twenties and thirties, at a time in their life when marriage and family start to take centre stage [1]. The reported annual incidence of rheumatoid arthritis between the ages of 18 and 34 years has been stated to be 8.7 per 100,000. This figure rises further up to 36.2 per 100,000 between the ages of 35 and 44 years [2]. Also, the prevalence of SLE in women in their childbearing years is around 1 in 500. In concordance, the prevalence of psoriasis is approximately 2–3% with almost 50% of these patients being women, of which many are in their childbearing age as the average age of diagnosis is 28 years and approximately 75% of cases occur before the age of 40 [3, 4]. Having an understanding of the reproductive health-related problems and being able to address them is critical for health professionals engaged in their care. For women who live with a chronic disease like inflammatory arthritis, this usually joyful experience of planning for a family may raise a number of queries, uncertainties, challenges and negative thoughts. As a result, important decisions need to be taken when planning a family. This reaches further than their ability to conceive, to include queries about the heritability of the disease, ability to maintain successful pregnancy, effect on the foetus, outcome of the pregnancy as well as risks of the medication on their baby. There is a clear need for specialized support as psychological factors can play an important role and may include a sense of guilt, stigmatization and loneliness with self-concern about their physical and functional ability to be a *mom* and whether they will be able to look after their children and family as well as care for themselves.

New disease-modifying antirheumatic drugs (DMARDs) and biologic therapy agents have shifted the management of inflammatory arthritis toward earlier, more aggressive therapy, with the ultimate goal of achieving full remission of the disease activity preventing structural joint damage. Alongside this, new treatment approaches, such as “treat to target”, have greatly improved treatment outcomes such as better functional ability and quality of life. These developments in the treatment paradigms have strengthened opinions that successful and safe pregnancies are possible, especially if pregnancy planning and screening for maternal and foetal risks are considered and implemented in standard practice, and the pregnancy takes place, while the disease is well controlled [5].

The introduction of specialized clinics for women with rheumatic conditions would enhance their care during pregnancy ensuring appropriate outcomes for both mothers and their baby. However, there has been an educational gap regarding how to set up such clinic. Based on our previous experience in setting up nurse-led early arthritis clinic [6], this service has adopted a similar approach, i.e. setting up a rheumatology nurse-led pregnancy clinic which would facilitate the provision of a holistic approach to both men and women in the childbearing period who would like to have a family. This article will present the unmet needs for such service, practical considerations in standard practice and targets and challenges that might face this model of care.

## Why it is important to have a pregnancy clinic for arthritic patients

Although rheumatologists are exclusively qualified to manage women living with ARDs during pregnancy and are generally familiar with the teratogenic potential of certain antirheumatic medications commonly used in standard rheumatology practice, a survey carried out by Chakravarty et al. found only 56% of rheumatologists included in the survey noted that routine family planning counselling was given to reproductive age women [7]. This may be because some rheumatologists do not consider family planning to be a part of their clinical responsibilities or they may see this as a burden with other competing priorities which need addressing during clinic consultations. Some may consider themselves under qualified or feel uncomfortable with discussing reproductive health issues, and this might reflect inadequate training regarding ways to initiate conversations about family planning or with prescribing appropriate contraception. On the other hand, while primary care physicians and obstetrician-gynaecologists have greater experience with family planning, they may be unmindful of the fact that both contraceptives and pregnancy could be linked to a flare of the rheumatic disease activity or that certain antirheumatic medications may affect foetal development. A survey carried out by Toomey and Waldron [8] found that only 8% of the primary care physicians who shared in the study felt that they had the expertise to provide family planning for inflammatory bowel disease patients, and in 57% of cases, they deferred family planning matters to subspecialists. It has also been found that some providers believe that the responsibility for family planning and teratogenic medication risk counselling of rheumatic disease patients should fall to the rheumatologists [9].

While on one hand, there are several challenges to consider when planning setting up this service; on the other hand, there are several factors which highlight the unmet needs of this group of patients and the urgent necessity to set up these clinics. These include the following: (1) It is known that there are associated risks to both the mother and the foetus in pregnancy in women living with ARDs. (2) With proper planning and careful management of the disease, such risks can be minimized. (3) There is a need for joint collaboration between the specialist physicians who are involved in the patients’ care. (4) More open discussions should take place with patients about their plans for having a family which should be made a priority as well as discussing the potential complications of pregnancy. (5) Experienced rheumatologists or rheumatology nurse specialists would be the best people to tackle this challenge. Therefore, appropriate consideration of both the short- and long-term goals is vital to ensure favourable pregnancy outcomes for both the mothers and babies.

To simplify the proposed service, it will be split into 3 phases:

## Planning for pregnancy: Patient-centred ethos

A range of family planning, pregnancy, and early parenting issues are raised in women of reproductive age who are affected by ARDs. [9]. Nearly half of pregnancies in Britain are not planned [Buyon et al., 2015]. This raises concerns in patients with ARDs as both the inflammatory condition as well as its treatments can cause problems with fertility, complications during pregnancy, disease activity and impact on contraceptive choices [10, 11]. Once diagnosed and as the patients, whether men or women, are being informed about the disease, its impact on their life, the approach to management and expected outcomes and their personal plans on the short and intermediate terms should also be discussed. Using a flexible narrative approach can encourage them to talk in their own words about their “lived experiences”, helping them to focus on what is important to them. It is likely that in their first visit, their main attention is on their arthritic condition. Later, as the arthritic condition is controlled, priority may shift to wanting to start or extend their family while on treatment. For this reason, regular assessment of the individual patient’s plans is important.

The stage of planning for pregnancy can be stratified into three different steps:

### Planning the pregnancy

#### Identification in the clinic

Regular assessment at each clinic visit is needed to identify patients who are considering starting or extending their family. This can be accomplished by using one of the patients reported outcome measures surveys, which the patient can complete prior to each visit [12]. The role of PROMs has now expanded from the static phase, capturing and measuring outcomes at a single point of time, to a more dynamic role aimed at driving their improvement. This does not only evaluate the quality of the inflammatory arthritis care provided but also assess their current health status, comorbidity, motivation and health-related quality of life [6].

#### Family planning counselling

This is particularly important for women with rheumatic diseases. Among women with SLE, RA and the inflammatory myopathies, well-controlled disease at the time of conception has been associated with better outcomes (e.g. normal birth weight and term deliveries) [13, 14]. On the other hand, in these conditions, poorly controlled disease at conception increases the risk of intrauterine growth restriction, caesarean section, preeclampsia and/or foetal loss [11]. For women with SLE, intensive preconception counselling and disease management have led to reduced disease flares with live birth rates similar to the general population [15]. These findings show that family planning may improve pregnancy outcomes through facilitating disease control prior to conception, as well as helping women whose preference is to avoid pregnancy altogether [16, 17].

#### Contraception counselling

Patients living with ARDs should have individualized contraception counselling, with open discussion taking place to agree the treatment targets, prioritizing the patient’s desires and future plans. As the disease is usually active, in the early stages, the primary target would be to control the disease activity. When the disease passes into a state of remission, it is at this time that pregnancy may become the priority. Contraceptive counselling is an integral constituent of the patient’s management at a certain stage when pregnancy needs to be prevented. Healthcare professionals running the pregnancy clinic should be aware of the principle categories of contraceptive methods and their safety profiles. Research evaluating contraceptive safety has mostly focused on SLE, RA and APS, whereas most methods appear to be safe for other rheumatic diseases.

When selecting the contraceptive approach is considered, special attention should be paid to reversibility, safety, convenience, non-contraceptive benefits, side effects and costs. Also, it should be tailored to the individual woman's preference. Efficacy of the contraceptive method selected is of particular importance to those patients whose disease may flare or are at increased risk of developing complication during pregnancy. Talabi et al. [18] reviewed the efficacy and safety of contraceptive methods in ARDs patients. Based on their efficacy, contraceptive methods can be stratified into 3 categories summarized in Table 1.

**Table 1:** Contraceptive tools for patients with autoimmune rheumatic diseases

Highly effective methods	Main features	Moderately effective methods	Main features	Least effective methods	Main Features
Progestin-only subdermal implants	The most effective contraceptives available (first-year failure rate 0.05%) Long acting: can provide contraception for up to 5-years Treatment safe with active SLE, APS, thrombosis Reversible: rapid return to fertility May cause irregular periods	Combined hormonal contraceptives, which contain both oestrogen and progestin (e.g. pills, patch, and vaginal ring)	Moderately effective (7/100) Pill (daily), patch (weekly), ring (monthly) Safe for most women with ARDs, including quiescent SLE Contraindicated if active SLE, history of APS, or thrombosis Reversible: rapid return to fertility Avoid if: age ≥ 35 years and cigarette smoking, history of breast cancer, severe hypertension, migraine with aura; history of endometrial cancers, stroke, or cardiovascular disease Side effects: nausea, breast tenderness, spotting for first few month When progestin-only pills are taken at the same time daily, efficacy is similar to oestrogen-containing methods	Male and female condoms	Failure rate: Female: 21/10; male: 18/100 Use PRN: only with sex Safe for all patients with ARDs, no hormones; reduces transmission of STIs; no prescription required Side effect/ contraindication: allergic reaction
Intrauterine devices (IUDs)	Highly effective (< 1/100) Long acting: provides contraception for up to 7 years Copper IUDs are hormone free and provide about 12 years of contraception Safe for women with ARDs, even those who are immuno-suppressed Safe with active SLE, APS, thrombosis Reversible: rapid return to fertility	Depot medroxyprogesterone acetate (DMPA)	Moderately effective (4/100) Short acting: short every 3 months Safe with active SLE, APS, thrombosis Reversibility: 10 months (median) Causes transient decrease in BMD, weight gain	Diaphragm	Failure rate: 12/100 Use PRN: only with sex Safe for all patients with ARDs, no hormones; reduces transmission of STIs; no prescription required Side effect/contraindication: allergic reaction
Female/male sterilization	For patients who achieved their desired family size Effective (< 1/100) Irreversible Possible side effects: Pain, bleeding, infection, surgical complications				

*SLE* systemic lupus erythematosus, *APS* anti-phospholipid syndrome, *BMD* bone mineral density

Rapid return to fertility means most women are able to become pregnant within several menstrual cycles after cessation of method [20]

Pregnancies per 100 women in first year of use [21, 22]

Providers should remember that pregnancy increases thrombotic risks more than any contraceptive method [23]

An alternative may be emergency over the counter contraceptives, preventing pregnancy up to 5 days after unprotected sex, e.g. progestin-only contraceptives; however, it was reported that its efficacy wanes by the day. Therefore, other prescribed emergency contraceptive pills, particularly for over-weight women, may be more reliable in preventing pregnancy within 5-days of unprotected sex. Nevertheless, the most effective emergency contraceptive is a copper IUD placed within 7-days of unprotected sex [19].

## Pregnancy

### Fertility

A high degree of collaboration between the reproductive medicine specialist, high-risk obstetrician and rheumatologist is needed when addressing fertility issues in ARDs patients. Such collaboration between these specialities maximizes the potential for a successful outcome while, on the other hand, minimizing maternal risk.

Earlier studies have shown that women with polyarthritis such as RA and SLE tend to have smaller families than do control groups [24]. The Danish national birth cohort between 1996 and 2002 found that pregnant women enrolled in the cohort with prevalent RA (onset before conception) were more likely to have had treatment for infertility (9.8% vs 7.6%) or to have taken months to conceive (25.0% vs 15.6%) [25]. Out of 245 patients in the PARA study in the Netherlands which included women who were pregnant or attempting to become pregnant, 205 (84%) became pregnant, while 64 (31%) had a time to pregnancy over 12 months. This appears to be due to multifactorial aetiology including disease activity, the direct impact of such disorders on fertility and certain medication exposure including preconception use of nonsteroidal anti-inflammatory drugs (NSAIDs) and prednisone (> 7.5 mg/day) or cyclophosphamide in SLE patients which diminishes the ovarian reserve. Other data, in RA patients, showed that time to pregnancy was not found to be associated with rheumatoid factor (RF) or anti-citrullinated protein antibody status or disease duration [26].

### Fertility preservation

Although the focus is on preservation of fertility by limiting use of cytotoxic medications when possible, in particular in SLE patients, and protecting the ovaries throughout cytotoxic therapy, this may be superseded by the need for prompt and effective treatment in severe disease. Cryopreservation of oocytes or embryos can be an effective option for preservation of fertility; however, this requires ovarian stimulation, and this might be impractical given the usual need to institute therapy quickly to prevent damage. There is also the risk of hyper stimulation in an already active SLE patient [27].

### Assisted reproduction techniques

These techniques include ovarian induction (OI) with or without in vitro fertilization (IVF) and embryo transfer. These techniques raise particular concerns for SLE patients, as ovarian hyper stimulation syndrome (OHSS) is a complication of IVF which results in a diffuse capillary leak syndrome with pleural effusion and ascites. This raised issues of potential relevance for SLE patients [28, 29].

#### Managing disease course during pregnancy

Discussing the impact of pregnancy on disease activity is important as this forms a basis for treatment recommendation. The patient condition needs to be well controlled and stable for at least 3–6 months before conception. Pregnancy can impact on the disease course in different ways which vary from one disease to another. Improvement in RA disease activity during pregnancy has been documented [30, 31]. However, during pregnancy, there are limitations in using the conventional measures of disease activity assessment as these measures may be confounded by other pregnancy-related symptoms. A study comparing different disease activity scoring tools in RA versus healthy controls during pregnancy found that DAS28-CRP without assessment of global health was the preferred tool during pregnancy for measuring RA disease activity [32].

It has been demonstrated that when using disability measures such as health assessment questionnaire (HAQ) during pregnancy, these measures decrease in the third trimester in comparison to its outcome scored immediately before pregnancy. Interestingly assessment of the pain score over the course of pregnancy revealed that there has been significant improvement in the pain measure with 60% of the women reported improvement, whereas only 19% described worsening. However, only 16% of the patients reported remission during pregnancy (defined as no swollen joints and no use of medications) [31]. One study reported reduction of the DAS-28 during pregnancy, in spite of the fact that over one-third of women were not receiving any medications specific for RA in the third trimester [30].

In the postpartum setting, disease activity has been reported to get worse more often. This has been shown in assessing different parameters of disease activity including joint counts, pain measures, and DAS [31]. In the PARA cohort, 36% of women had a moderate flare and an additional 4% a severe flare [30].

In SLE, the risk of flare up of the disease activity during pregnancy is one of the major problems. Earlier studies revealed variable flare rates of flare ups which ranges between 25 and 65% [33, 34]. This disparity in the flare up of the disease activity during pregnancy extends to include variable responses at the different organ/systems level; e.g. musculoskeletal flares are less common, while renal and hematologic flares are more common. The majority of the flares in pregnancy are mild-to-moderate, with only small percentage of patients developing severe flares [34]. Predictors of disease flare which showed significant increase of flares risk in SLE women during the pregnancy include active disease during the 6 months prior to conception, history of lupus nephritis and discontinuation of antimalarial medication [35]. Table 2 shows a protocol for anti-natal monitoring the ARDs patients during pregnancy.

**Table 2:** A protocol for anti-natal monitoring the autoimmune rheumatic diseases patients during pregnancy

Clinical assessment	Measurements and investigations	Specific monitoring
Rheumatology clinic: 4–6 weekly, more frequent if the disease becomes active or flares	Standard: Each visit: blood pressure, body weight Full blood count, serum uric acid, liver functions, urea, creatinine, electrolyte levels, urinalysis SLE patients: protein/creatinine ratio, complement levels and dsDNA antibodies	Positive anti-Ro antibodies: foetal echocardiography, weekly from week 16–26 and biweekly thereafter, continuing till delivery
Obstetrician: monthly till week 20, then 2 weekly till week 28, and weekly thereafter	Ultrasound: -early pregnancy for gestational dating, -between week 16–20 to screen for foetal anomalies, −4 weekly thereafter to monitor growth	Preeclampsia: uterine artery Doppler study (week 20 and 4 weekly thereafter), foetal umbilical artery Doppler velocimetry (weekly from week 26 onwards)
	Foetal surveillance tests (FST): weekly starting form week 26	Intra-uterine growth retardation (IUGR): increase frequency of growth monitoring by ultrasound and FST

*FST* foetal surveillance tests, *IUGR* intra-uterine growth retardation

#### Pregnancy outcomes

It has been demonstrated across multiple cohorts and wide ranging geographical locations that delivery by caesarean section is more common among women with ARDs [36, 37]. Women who had moderate-to-high disease activity were more likely to have caesarean section in comparison to those who have low disease activity [38].

Increased risk of preeclampsia has been demonstrated in some studies among rheumatoid arthritis women [39]; however, this was not confirmed in other studies [40–42]. This variation of studies outcomes might be attributed to different patient populations or preeclampsia case ascertainment. In SLE patients, it might be difficult to differentiate between lupus nephritis flares and preeclampsia; as in both conditions, deteriorating renal function and increasing proteinuria, hypertension and thrombocytopenia may occur. Table 3 shows an approach to distinguish between the 2 problems. Investigation wise, a higher risk of preeclampsia and poor obstetric outcomes was associated with abnormal uterine artery waveforms [43–45].

**Table 3:** How to differentiate between preeclampsia and lupus nephritis in SLE patients

	Preeclampsia	Lupus nephritis
Clinical		
Blood pressure: hypertension	After 20 weeks of gestation	Any time during pregnancy
Other organ affection	Occasionally CNS	Evidence of non-renal active SLE
Laboratory investigations		
Standard blood testing		
Platelets	Low–normal	Low–normal
Creatinine	Normal–raised	Normal to raised
Uric acid	Elevated	Normal
Immunology testing		
Complements	Normal–low	Low
Anti-dsDNA	Absent or unchanged	Rising titers
Urine testing		
Urinary sediment	Inactive (uniform pattern, reflect renal damage, no correlation with clinical course)	Active (urine sediment reflect lupus nephritis histopathology)
24-h urine calcium	< 195 mg/dl	> 195 mg/dl
Management:		
response to steroid therapy	No response	Good response

Although several studies demonstrated an increased risk of preterm births [46, 47], this was not confirmed in other pregnancy outcomes research [35]. Interestingly, prematurity was associated with increased HAQ values during pregnancy. Variable data have been published regarding the impact of the disease on the infant weight. Low birth weight was reported in RA patients in some research, whereas other studies did not report this [48].

#### Medication Counselling

From pre conception, through pregnancy and following delivery, management decisions are complex due to the lack of data and the potential for teratogenicity of the therapies available. Standard and biologic disease-modifying medications, as well as corticosteroids in pregnancy, have been reviewed for their compatibility and safety [49]. For patients living with inflammatory arthritis, who are considering starting a family, their treating rheumatologist/ rheumatology nurse are the best source of information and support. Before considering getting pregnant, patients must be in remission, achieved by using DMARDs and biologics to control the disease activity. The aim should be to have an individualized treatment plan achieved by appropriate counselling regarding the risks and benefits of these medications optimizing the chance of a healthy pregnancy and baby.

## Breastfeeding and postpartum care

Consideration needs to be given to the disease activity, the need for medication and the health benefits of breastfeeding when making a decision of whether or not to breastfeed. This decision should be made for each patient on an individual basis. Worse disease activity in first time breastfeeding women at 6 months postpartum was noted in a prospective study compared to non-breastfeeding women in the same time frame [50]. Prednisolone appears to be a suitable option for breastfeeding mothers who sustain a flare of their RA. The levels of prednisolone in breast milk reach 5–25% serum levels, with an estimated 0.1% of the mother’s dose being absorbed by the infant and insignificant amount compared to the endogenous production [51].

Both the BSR [49] and the American Academy of Paediatrics [52] advised that NSAIDs such as ibuprofen, diclofenac, indomethacin, naproxen and piroxicam are compatible with breastfeeding. A good option is ibuprofen due to its low rate of transfer, short half-life and low levels reached in breast milk [53]. The BSR has published a resource to help guide clinicians and patients regarding the safety of medications which can be used while breastfeeding [49].

### Vaccination of the newborn

Given the fact that IgG antibodies are able to cross the placenta in the third trimester, with the exception of certolizumab, anti-TNF biologics have been found to be detectable in babies up to 6 months old of mothers treated with biologics [54]. Live attenuated vaccines should therefore be avoided based on this data, in babies up to 6 months old whose mothers have been exposed to biologics during the second half of pregnancy [55–60]. Although data is available regarding the lack of certolizumab transfer to cord blood, this is limited to a small number of patients. Also, no data regarding live vaccination of newborn of mothers treated with certolizumab has been published [61, 62].

The fatal case of a newborn, with disseminated tuberculosis exposed to infliximab, who was vaccinated with vaccinated with Bacillus Calmette–Guérin (BCG) vaccine, highlights the importance of avoiding live-attenuating vaccines during at least the first 6 months of life [63, 64]. EULAR suggests points to consider for using antirheumatic medications, before pregnancy, as well as during pregnancy and breastfeeding. Only babies exposed to biologics before 22 weeks can, according to standard protocols, receive vaccines including live vaccination. Although babies exposed to biologics during the second and third trimester can follow the vaccination programme, they should not receive live vaccines of the first 6 months of life. Measures of the biologic in question, in the child serum, may guide the decision as to whether or not give live vaccination [65].

**Fig. 1:**
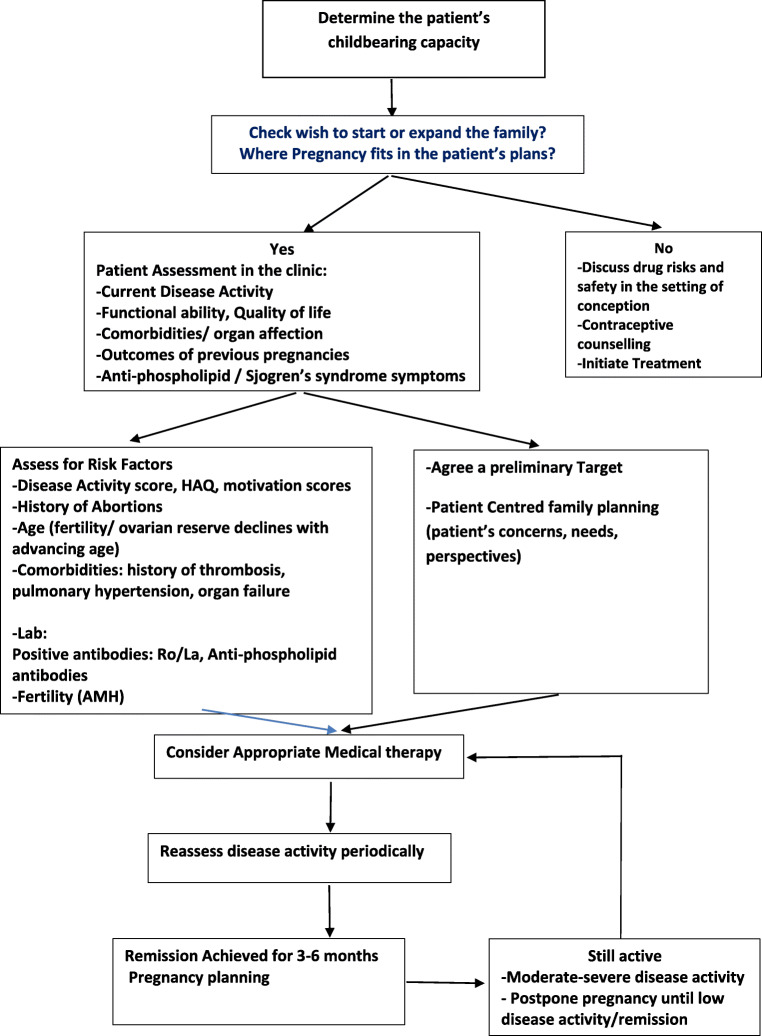
Flow chart showing the set-up of the pregnancy clinic and how to manage family planning for women living with rheumatic diseases

In conclusion, rheumatologists must lead family planning for women living with ARDs. A good option for patients to receive counselling and to be able to develop individualized care plans are rheumatology nurse-led pregnancy clinic. Such clinics can provide extensive monitoring and education, helping patients toward the best options for themselves and their newborn babies. Figure 1 shows a model of the rheumatology nurse-led pregnancy clinic service.
